# Notes and News

**Published:** 1898-06-25

**Authors:** 


### NOTES AND NEWS.

(Collected and Communicated.)

The plague in Hong Kong is rapidly decreasing.

Mr. Chisholm Williams will lecture on "Lateral Curvature of Spine" at the City Orthopædic Hospital, on Tuesday, June 28th, at 5.30 p.m.

The North London Hospital for Consumption has received a donation of £1,000, to endow a bed in the hospital, from "R. S. P.," per Canon Fleming.

An anonymous subscriber has sent £2,000 to the Lord Mayor of Liverpool to be devoted to a city charity. The Mayor has given it to the building fund of the Stanley Hospital.

Sir James Clerk-Rattray, K.C.B., of Craighall, Blairgowrie, has given £1,000 towards the local cottage hospital scheme which was started in commemoration of the Queen's Diamond Jubilee.

The College of Physicans of Philadelphia announces that the sum of five hundred dollars will be awarded to the author of the best essay for the first Nathan Lewis Hatfield prize for original research in medicine. The subject is: "A Pathological and Clinical Study of the Thymus Gland and its Relations." The competition will be open to members of the medical profession and men of science in the United States.

It has been decided by the governors of The London Hospital to proceed with the following improvements in the hospital at once: The receiving room is to be enlarged by an extension into the front courtway, and a certain rearrangement of the offices abutting thereon will be made. A proper suite of operating rooms, consisting of two theatres, two operating rooms, anæsthetising rooms, and instrument and sterilising rooms, is to be built, the cost having been generously provided by an anonymous donor. The present old plaster on the walls of the wards is to be replaced by a new cement facing, and in other respects the wards are to be modernised. More rooms for the residents are to be built, the top wards improved, and accommodation for more nurses provided.

The Countess of Gosford has accepted the position of Patroness of the St. Monica's Home for Sick Children in succession to H.R.H. the late Duchess of Teck.

A very elaborate paper on the statistics of deaths in childbirth was read before the Royal Statistical Society on June 21st by Mr. T. A. Coghlan. In regard to the mortality of first confinements, it appears that in married women the safest ages are the twenty-second and the twenty-third years. In unmarried women the minimum risk occurs between 25 and 30 years of age. It is, however, to be noted that statistically the risk of death in a first confinement is at every age greater for the unmarried than for the married. Taking the whole mass of confinements, however, and comparing their fatality at different ages, it appears that on an average 1 woman out of 23 who marries at age 20 will die in childbirth; 1 in 32 at age 25; 1 in 39 at age 30; 1 in 43 at age 35; and 1 in 42 at age 40.

Writing on the coming election to the Council of the Royal College of Surgeons, an F.R.C.S. urges that the electors ought to know the opinions of those for whom they are asked to vote. It has been urged, he says, that it is undignified for candidates for the Council to canvass for support on account of any special line of policy, as the selection depends rather upon their professional eminence. Against this he urges that professional eminence by no means implies "sound medical policy," and that candidates for other responsible appointments do not expect to be elected without some general indication of the line of policy which they intend to take. It is interesting to consider, although it is a little difficult to imagine, what the Council of the Royal College of Surgeons would be like if its members were all appointed for their prominence in what are sometimes called medical politics, instead of for their professional eminence.

The *Press Bazaar News* will be the smallest evening paper ever issued—and at the highest price, viz., one shilling. But it will be a curiosity—a thing to buy, and a thing to keep—for we may well ask, When again are we likely to find such a combination of journalism of every colour engaged in the production of one little print? In many ways this paper will be something quite out of the common way. It will be a real evening paper, containing all the news, but its staff will combine the editors of almost every important paper in England. Lords and ladies, preachers, actors, men of science, and men of business will contribute to its special departments. It will be set by the linotype machine, and all the metal used will be melted by electricity, for no naked lights will be allowed, and it will be printed by hand. To those who attend the bazaar the production of this unique newspaper will be a "show" of the greatest interest, while to the large number who cannot be there we feel sure that a copy of such a newspaper will be an interesting memento of a most interesting event. A copy of each day's issue will be sent on remitting 2s. 6d. to Arthur H. Pollen, 188, Fleet Street, E.C. We should add that every penny received for the sale of the paper will go to the charity, as the Linotype Company is standing the whole expense.

